# A Cross-Temporal Meta-Analysis of Changes in Left-Behind Children’s Mental Health in China

**DOI:** 10.3390/children9040464

**Published:** 2022-03-25

**Authors:** Xiaoyi Zhang, Zhoudao Dai, Collins Opoku Antwi, Jun Ren

**Affiliations:** 1 Key Laboratory of Intelligent Education Technology and Application of Zhejiang Province, Zhejiang Normal University, Jinhua 321004, China; 201810100010@zjnu.edu.cn (X.Z.); cantwi28@zjnu.edu.cn (C.O.A.); 2 School of Education Science, Huangshan University, Huangshan 245041, China; zxy@hsu.edu.cn; 3 College of Teacher Education, Zhejiang Normal University, Jinhua 321004, China

**Keywords:** (non-) left-behind children, mental health status, mental health test, cross-temporal meta-analysis

## Abstract

A considerable body of research using the Mental Health Test (MHT) has explored the psychological repercussions of the physical separation of children from one or both parents as they pursue better economic prospects in cities. Generally, these studies compare the mental health status (MHS) between left-behind children (LBC) and non-left-behind children (NLBC). That notwithstanding, little is known about the real policy impact of these studies on the ground over the years. Using a relevant search strategy and selection criteria, we identified qualified studies (N = 102: 2004 to 2019). Cross-temporal meta-analysis (CTMA) was performed on these studies for dynamic trends. Our results demonstrate: (1) a slight but significant change in MHS of both LBC and NLBC, with LBC being significantly worse off over time; (2) a significant deterioration in MHS of LBC over time, particularly among left-behind boys (LBBs); (3) a stable and significant decline in MHS of left-behind junior high and elementary school students, respectively; and (4) a more substantial degradation in MHS of LBC with both parents absent compared with LBC with a parent present. The findings reveal that the efforts of, and collaboration among, researchers, policy experts and politicians are producing results. Nevertheless, more targeted research is needed to unearth the underlying issues that generate the differences among subpopulations of LBC to better inform pragmatic interventions for collective psychological wellness of LBC.

## 1. Introduction

The great rural-urban migration in the wake of the impressive economic rise of China has created the phenomenon labelled as the “left-behind children (LBC)”. The term refers to minors under the age of 18 who are separated from one or both parents because the parent(s) seeks greener pastures in urban centers [1]. One might ask: “why not come along with these children or bring them over in a short time after the parents settle in the cities?”. Migrants in big cities from rural areas have no household registration (called Hu Kou in China) and therefore lack access to social facilities, including education and health care in their new homes. As a consequence, millions of children have been left behind in rural China. China’s Ministry of Education estimated that about 12.897 million children were left behind [2]. One can imagine that the adaptation challenges that these children face in various facets of their development are significant considering the salient role of parents in children’s socialization within the family unit and in other relevant domains of life, such as schooling. A number of studies have sounded the alarm bells on the socio-developmental, emotional, educational and mental health implications of not having one or both parents in the life of children. In particular, these children are subjected to malnutrition, various forms of neglect and an enormous emotional burden of the fear of missing out, and this fear is particularly intense as children reach their senior grades [3,4]. The mental health of LBC has always been the core issue of academic and policy concern, and rightly so. The mind, as they say, is the engine of growth. For example, the word “mental health” ranks fourth in the LBC corpus in China’s How Net. The issue of LBC is so critical that it received, deservedly, a special treatment in China’s development goals. On 27 September 2021, the State Council of China issued the outline of China Children Development Plan (2021–2030). This plan emphasizes care for and protection of LBC and other vulnerable groups in rural China and children in distress overall as critical foci of the Chinese Government, hinting at the enormity of the issues of LBC and the government’s commitment in this regard. 

This and other commitments are in order for dealing with the issue of LBC and their welfare. The contributions of research to the policy developments tailored to address the psycho-emotional, physiological and educational challenges of LBC cannot be overstated. Yet, the temporal reflection of these impacts in subsequent studies have not been undertaken. Thus, due to the policy implication of research on mental health status (MHS) of LBC, do the levels or degree to which physical separation impacts the MHS of LBC decrease in subsequent research, reflecting policy effects of prior research? Put differently, we expect that initial research culminating in policy developments would induce behavioral and lifestyle changes at the micro level of guardianship (thus creating developmental behaviors of those who stay with LBC) and of departed parent(s) (e.g., ensuring frequent check-ins using modern ICT tools such as QQ and WeChat). In other words, do research-informed practical or policy interventions fostering socio-cultural and techno-economic changes over time have a decreasing or increasing effect on the MHS of LBC? This study seeks to explicate these complex dynamics with cross-temporal meta-analysis (CTMA) of the link between publication year and Mental Health Index (MHI) reported in papers. 

When measuring the MHS of LBC, previous studies have generally adopted the following measurement tools (with samples mainly from primary and middle school students): the Mental Health Test (MHT), SCL-90 scale and Middle School Students’ Mental Health scale. The content of these scales varies. For example, SCL-90 and Middle School Students’ Mental Health scale have been found unsuitable for measuring the MHS of primary school students [5]. However, LBC are distributed across all age groups below 18 years. Therefore, we meta-analyze studies that employed the MHT, which is applicable to the age range of LBC and is therefore used frequently as the mental health assessment tool for LBC, to dynamically model the relation between the publication period of the papers and the MHI. Moreover, the MHT covers a comprehensive spectrum of psychological malaise. 

The need to map out the increasing or decreasing MHI through time is urgent for two reasons. First, some meta-analyses on MHS of LBC show a deteriorating trend in the MHS of LBC when compared to the non-left-behind children (NLBC) [6,7]. However, conventional meta-analytic techniques are ineffective in dealing with the “age effect” of psychological variables [8]. To this effect, traditional meta-analytic studies do not include publication year, failing to account for the socio-cultural and techno-economic milieu of the meta-analyzed studies. To address this challenge, we adopt the CTMA method, which has the capacity to reveal large-scale differences in time by isolating research in chronological order as a way of dealing with the “age effect” in these published materials [9]. Second, we assess the changing trend in the MHS of the LBC across a relevant subpopulation to properly tease out targeted policy interventions that are not one-size-fits-all, if indeed MHS is generally increasing with time. Accordingly, we compare the changes in MHS of LBC across gender, grade and guardianship type. Additionally, we explore the changes in MHS of all LBC and NLBC across time. 

## 2. Materials and Methods

### 2.1. Research Tools: The Mental Health Test (MHT)

The MHT utilized in this study is a standardized mental health diagnostic scale revised by Zhou Bucheng [10]. This scale is suitable for fourth-grade primary school to third-grade junior high school students in China. It consists of 100 questions and is divided into eight dimensions: learning anxiety, social anxiety, loneliness anxiety, self-blame tendency, allergic tendency, physical symptoms, terror tendency and impulse tendency. The students respond with a “yes” or “no”, with “yes” scoring 1 point and “no” scoring 0 points. The higher the score, the worse the mental health level. The split-half reliability of the whole scale is 0.92, and the structural validity is higher than 0.510.

### 2.2. Literature Collection Methods

The researchers searched the three Chinese databases—CNKI Database, WANFANG Database and VIP Database—by matching any fields of “Left-behind Children”, “Left-behind Children” with “Mental Health”, “Mental Health Test” and “MHT”. A total of 10,031 documents were retrieved. By reading the titles and abstracts, 9451 articles were excluded, and the remaining 580 articles were further examined for suitability. Finally, 102 papers were included in the study. The screening criteria are as follows: (1) the tool for measuring mental health is MHT; (2) the research subjects were LBC in mainland China; (3) the study at least stated the sample size as well as the average and standard deviation of each index; (4) excluded special groups as subjects, such as LBC in earthquake-stricken areas and disabled LBC; (5) in cases of duplicate substance (thus, different documents published by the same author using the same data), we used the earliest and most complete documents. 

### 2.3. Document Coding

In order to facilitate the use of CTMA, we followed the protocols described in previous research [11] for the studies’ coding. Like the study prior, the publication year minus 2 is regarded as the data collection time for documents that did not report data collection time. All the research data were coded according to sample size, gender, education period, guardianship type and other indicators (see Table 1). Whether or not a child was left behind, the gender, school period and type of guardianship of the LBC were analyzed as subdomains of LBC research. Data group 1 and sample size 1 in Table 1 were used for the CTMA, and data group 2 and sample size 2, also in Table 1, were used for ordinary meta-analysis. Please note that the sample used here does not include LBC in senior middle schools. Following the screening criteria in 2.2, the total number of studies that qualified were classified under data group 1 and against the various subpopulations with a corresponding amalgamated sample size under sample size 1. To attain the temporal change differences between LBC and NLBC, we further sorted the studies under data group 1 by checking whether or not they compared LBC with NLBC. All the comparative studies were reclassified under data group 2 with their combined sample sizes under sample size 2. Therefore, data group 1 was used for analyzing the overall temporal change score in MHS of both LBC and NLBC whilst data group 2 was used to map out the temporal changes between LBC and NLBC across subpopulations such as gender.

### 2.4. Data Analysis

Microsoft Excel 2010 and IBM SPSS version 22 were used for the processing and analysis of valid data. The formula for the calculation of the effect d is: d = (x_2004_ − x_2020_)/SD, and the coefficient of determination r^2^ = d^2^/(d^2^ +16). The difference was statistically significant with *p* < 0.05.

## 3. Results

### 3.1. Overall Change in MHS of LBC and NLBC with Time

**Figure 1:**
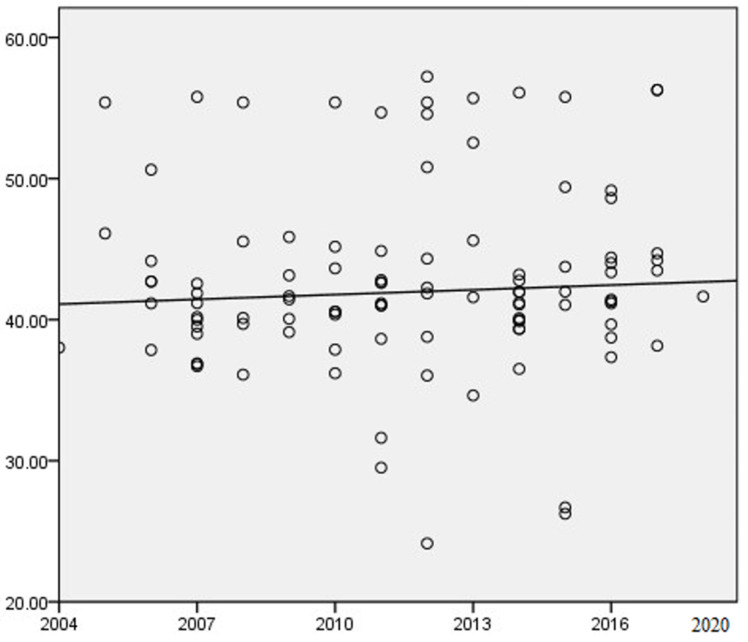
Changes in mental health scores of left-behind children over the years.

In order to visually display the changes in MHS with the years, taking the year of data collection (YoD or age) as the abscissa, MHT was divided into ordinate to draw a scatter chart. Figure 1 shows that the mental health score of LBC and NLBC in China increases slowly with the years.

In order to describe more accurately the changes in mental health scores for LBC and NLBC with the years, we controlled for sample size in the correlation and linear regression analyses of the mean value of MHT indexes and age (see Table 2). Results from the correlation analysis showed that whilst scores of learning, social anxiety and loneliness positively correlated with age to a significant degree, those of physical symptoms, terror, self-blame, allergic and impulsive tendencies were negatively correlated with age. Further assessment of the results indicates that only LBC’s loneliness tendency is positively correlated with age; however, their physical symptoms, learning and social anxiety, as well as self-blame and allergic tendencies, are all significantly negatively correlated with age. The standard deviations (SDs) of LBC’s MHT domain scores were significantly positively correlated with age except terror tendency. On NLBC, their MHT domain scores’ SDs were significantly negatively correlated with age, except allergic tendency. Moreover, the coefficients of the regression analyses of the eight indicators of MHT were all significant for both LBC and NLBC, and that age can explain 0–3.7% of the eight indicators for LBC and 0–5.8% for NLBC.

### 3.2. Changes in MHS of LBC and NLBC with Time

To investigate changes and differences in mental health scores between LBC and NLBC, we calculated the effect size d [12] of the changes in MHS of the two subpopulations. The results show that the average effects for three of the eight factors of MHT for LBC were negative, whilst those for the other five factors were positive. Among these, the average D value for loneliness anxiety is 0.51 (moderate effect). The overall average D value of 0.014 for all the eight factors failed to achieve a small effect, suggesting that the mental health of LBC did not change much with the years. With NLBC subpopulation, five factors recorded negative mean effects, three with positive values. Among these, the D values for loneliness anxiety, self-blame and terror tendencies were 0.49, −0.30 and −0.29 (all small effect). However, the D value for learning anxiety was −0.69 (moderate effect). The overall average D value for all eight factors was −0.134 (small effect unattained). These results show that the mental health level of NLBC increases with the years but is not statistically significant (see Table 3).

To tease out the effect of being “left-behind” on LBC’s mental health, the study used an ordinary meta-analysis method, with LBC as the experimental group and NLBC as the control group, and calculated D of their average effects. The results showed that all other indicators except self-blame had a small effect (between 0.2 and 0.5), indicating that, compared with NLBC, the mental health level of NLBC is poorer across the eight dimensions of the MHT (see Table 4).

### 3.3. Changes in MHS of LBC across Gender with Time 

To obtain a better appreciation of gender differences in mental health experience of LBC across time, we meta-analyzed the data with complete gender information (see Table 5). The results show that the average effects of six factors of left-behind boys were positive, while those of two factors were negative. Moreover, the D values of learning anxiety and terror tendency were 0.36 and 0.27 (all small effect), while the average D value of all the eight factors was 0.170, suggesting the absence of even a small effect. This shows a decline in the mental health of left-behind boys with the years, but without being statistically significant. Left-behind girls recorded five positive factors and three negative factors. The D value for learning anxiety was 0.34 and loneliness tendency was 0.65, achieving small and moderate effects, respectively. However, the average D value for all the eight factors was 0.114 (small effect unattained). This also shows that, like the boys, the mental health level of left-behind girls declines with the years but is not statistically significant.

The difference in MHS between left-behind boys and left-behind girls was explored using ordinary meta-analytic technique. Taking left-behind boys as the experimental group and left-behind girls as the control group, we calculated Cohen D (see Table 6 for details). The results showed that the average effect of all indicators of MHT, except allergic tendency, was less than 0. Moreover, the average effect of learning anxiety, self-blame and terror tendencies and total score ranged from 0.2 to 0.5, indicating a small effect. The results show that the mental health level of left-behind boys is slightly better than that of left-behind girls.

### 3.4. Changes in MHS of LBC across Study Level with Time

To assess the extent of change in MHS through time across study levels of left-behind primary and middle school students, CTMA was conducted (see Table 7). The results show that, for left-behind primary school students, the average D values for seven factors were positive and one factor was negative. Thus, the D values for learning anxiety, social anxiety and allergic tendency were −0.49, 0.28 and 0.31 (all small effect). However, the D value for impulsive tendency was 0.62 (moderate effect), whilst the D value for loneliness tendency was 1.09 (large effect). The overall average D value of all the eight factors of MHT was 0.254 (small effect), indicating that the mental health level of left-behind primary school students is declining with the years. On the other hand, the Left-behind junior middle school students recorded four positive and four negative factors. The results show D values for loneliness, self-blame, physical symptoms and terror to be 0.35, −0.44, 0.38 and 0.20 (all small effect). The overall average D value for all the eight factors was −0.058 (small effect), suggesting that the mental health level of left-behind junior high school students record little change over the years.

To ascertain the significance of the differences in changes in MHS across LBC’s study levels, the average effect (d) was calculated by using traditional meta-analysis by specifying left-behind primary school students as the experimental group and left-behind junior high school students as the control group (see Table 8). The results show that there is little difference in mental health levels between left-behind primary school students and junior high school students, except for a small effect in impulsive tendency. 

### 3.5. Changes in MHS of LBC across Guardianship Type with Time

To explore the changes in the mental health level of LBC with different guardianship types—single-parent and lack of parental guardianship—we performed CTMA on the data related to guardianship type (see Table 9). The results showed that the average effects of five factors were positive for LBC under the guardianship of a parent, whilst the rest of the three factors were negative. The D values for learning anxiety, allergic and terror tendencies were 0.46, 0.33 and −0.39 (all moderate effects). The average D value for all the eight factors was 0.041, which shows that the mental health levels of LBC under single-parent guardianship changed little over the years. The LBC who lacked a parental guardianship recorded six factors with positive average effects and two with negative effects. The D values for anxiety, self-blame and terror tendencies were 0.34, 0.42 and −0.47 (all small effects), whilst those of loneliness and allergic tendencies were 0.72 and 0.60 (all moderate effects). The D value for learning anxiety recorded the largest effect (1.06). The overall average D values for the eight factors was 0.349, showing that the mental health levels of LBC who lacked parental guardianship declined with time.

We further explored the influence of guardianship types on LBC’s mental health by performing traditional meta-analysis. First, the LBC who lacked parental guardianship were classified as the experimental group, and the LBC with single-parent guardianship and NLBC (regarded as children with parent(s’) guardianship) taken as the control group. We then calculated the d1 and d2. Next, we took the LBC with single-parent guardianship as the experimental group and NLBC as the control group and then calculated d3 (see Table 10). The results showed that d1 ranged from 0.02 to 0.19 (small effect unattained), indicating that there was no significant difference in mental health levels between LBC with single parent guardianship and those without parental guardianship. In d2, all dimensions except self-blame tendency achieved small effect. This shows that the mental health level of LBC without parental supervision is lower than that of children with parental supervision. In d3, all dimensions except self-blame and terror tendencies, including the total score, attained a small effect, indicating that the mental health level of LBC under single-parent guardianship is lower than that of children under two-parent guardianship. 

## 4. Discussion

### 4.1. The Mental Health of LBC Has Improved over Time but the Negative Impact of Being “Left-Behind” on Mental Health Remains

This paper investigates the changes in MHS of LBC and NLBC in the past 16 years. The findings show that the scores of mental health of LBC and NLBC are significantly correlated with time. However, the difference between the observed changes through time across LBC and NLBC was nonsignificant. On the whole, the mental health levels of LBC and NLBC are progressively better (except for individual indicators). This is similar to other CTMA studies on the mental health levels of minors [13]. At the same time, the difference in mental health levels between LBC and NLBC is narrowing. These results may be related to the degree to which China attaches great importance to the mental health of elementary and middle school students. Over the years, a number of research-informed intervention policies have been enacted by the Chinese Government. These include the “Guideline for Mental Health Education in Primary and Secondary Schools” issued by the Ministry of Education of China [14], “The inter-ministerial joint conference system for the care and protection of LBC in rural areas and the protection of children in difficulties” issued under the leadership of the State Council of China [15] and the “Opinions of the State Council on Strengthening the Care and Protection of LBC in Rural Areas” issued by the State Council [16]. We can imply that these policies, informed by research, are making a positive difference in the mental health of LBC.

There are differences between LBC and NLBC across some indicators of mental health through time. The most significant one was found between learning anxiety and loneliness. Specifically, the learning anxiety of NLBC decreased significantly over time, while it increased slightly among LBC with time. Although both LBC and NLBC exhibit learning anxiety, NLBC are more likely to get timely support (including parental and peer support etc.). However, the same may not apply to LBC, who may even experience anxiety asking for support from guardians depending on their relation. Moreover, social support for children’s resilience has come up as relevant buffering element in cultivating children’s mental health. On a less positive note, a meta-analytic study of children’s resilience shows that LBC are lower in resilience than NLBC [17]. This may cause the LBC to experience negative events more intensely than the NLBC, making it even more difficult to adjust and recover [18]. Secondly, the loneliness tendency of LBC and NLBC increased with time. This should be taken seriously. Loneliness is an emotional reaction that diminishes one’s sense of community, making children lose trust in society and develop an impaired sense of belongingness and fellow feeling. Moreover, loneliness dampens sense of self, leading to a series of psychological problems [19]. Why is the loneliness of left-behind and non-left-behind children increasing year by year? On the one hand, primary and secondary school students are in a digital society, and the popularity of smart phones has cut off the opportunities for face-to-face communication between people [20]. On the other hand, the reluctance of primary and middle school students to communicate with their parents or other non-peers may also be the reason for the upward trend in loneliness [21]. One meta-analytic study shows that the mental health indicators of LBC are worse than those of NLBC, but they were small effects (between 0.2 and 0.5). This is consistent with the previous findings of another meta-analytic study [22]. That parents’ company and help are a profound loss in the lives of children, impacting their psychology and behavior [23]. That is, being “left-behind” really affects the mental health of LBC.

### 4.2. The Mental Health of Left-Behind Boys and Girls Shows a Downward Trend with Time

It is revealed that, on the whole, the mental health levels of both left-behind boys and left-behind girls shows a downward trend with time. However, the left-behind boys report a more significant decline, indicating that the evolving social and cultural context of the times have a greater influence on left-behind boys, which is similar to the previous research results of LBC’s resilience [24]. According to the results of the general meta-analysis, the mental health level of left-behind boys is better than that of left-behind girls. This shows that, compared with boys, staying behind has a greater negative impact on girls. Previous studies found that the mental health problems of left-behind girls are more serious than those of boys, and the scores of left-behind girls are significantly higher than left-behind boys in four factors: learning anxiety, self-blame, allergy and terror [25,26]. Studies have also shown that there are differences in the nutritional status of LBC of different genders, that is, staying behind has a significant negative impact on girls’ physical health [27]. Therefore, the left-behind girls are weaker than the left-behind boys in psychological and physiological aspects. Left-behind environment makes the living environment not supportive of the left-behind girls [28], and their mental health level gradually deteriorates.

### 4.3. The Mental Health Levels of Left-Behind Elementary School Students Have Declined Significantly with Age, While the Level of Left-Behind Junior High School Students Has Not Changed Much

The mental health level of left-behind primary school students is progressively declining and the change range is significantly higher than that of left-behind junior high school students. This may be because primary school students are younger, lack the ability to cope with setbacks and pressures [29] and have low self-regulation ability and poor psychological endurance [30]. Indeed, separation at a younger age was found to exert a more substantial influence on the decline of children’s life satisfaction [26]. In terms of learning anxiety, left-behind primary school students showed a downward trend, while that of left-behind junior high school students hardly changed. It may be because primary schools have no pressure to go to school, while junior high school students have to go through the increasingly competitive senior high school entrance examination. The results of meta-analysis showed that the mental health level of left-behind primary school students is lower than that of left-behind junior high school students, but the difference is not significant. However, there are also cross-sectional studies that show that the mental health level of left-behind primary school students is significantly lower than that of left-behind junior high school students [31]. Younger children have stronger attachment needs to their parents, and elementary school students’ cognitive and psychological development qualities are slightly worse than those of junior high school students. Facing the lack of parent-child needs, they are prone to mental health problems such as loneliness and anxiety.

### 4.4. The Mental Health of Left-Behind Children without Parental Supervision Significantly Declined over the Years

The mental health level of left-behind children who lack parental supervision continues to decline with time, while the mental health level of left-behind children under single-parent supervision does not change significantly over the years. This suggests that the lack of parental supervision is more harmful to children’s physical and mental development than that of single parent supervision. This is likely because parents are children’s first line of defense in life [32]. The results of general meta-analysis show that compared with children under parental supervision, the mental health of LBC under single-parent supervision and those without parental supervision is worse, and basically in the range of small effects. In addition, there is no difference in the mental health level between the left-behind children who are supervised by one parent and those who lack parental supervision. These findings collectively suggest that it is questionable to focus only on LBC whose parents go out to work or who have one of them who goes out to work, with the other incapable of guardianship [33].

### 4.5. Study Implication

Long-term attention needs to be paid to the mental health of left-behind children. For future educational practice it is very important to improve the mental health of left-behind children and overcome the negative effects of children being left behind. According to the unique psychological characteristics of left-behind children, they should be counseled in many aspects, such as cognition, interpersonal relationship, parent-child relationship, etc. Correct and positive professional psychological guidance should be made accessible to left-behind children to help improve their mental health.

The mental health of left-behind girls needs more attention. In future educational practice, attention should be paid to gender differences in the psychological development of left-behind children, and more gender-sensitive mental health education should be designed. For example, setting up a counseling course specifically for left-behind girls to show more confident and healthy role models for left-behind girls, teach them to resist unhealthy value orientations, nurture their self-esteem, self-love and prevent sexual harm. Parents should also give more care to their daughters, provide adequate parenting resources and ensure a balanced distribution of family resources for sons and daughters.

Whenever possible, parents should be encouraged not to leave their children when they are young. An early parent–child relationship has a decisive impact on children’s growth. Separation from parents at a young age and staying behind have a greater negative impact on children’s behavioral and emotional development. To reduce the parental impact of parent–child separation on children, parents should try to avoid leaving their children at home when they are young. Where children from families where one parent goes out to work and the other has guardianship ability, the child is not considered left-behind. Therefore, even though, according to the latest definition, such a child (or children) is not left behind, our results show they still need more attention to their physical and mental development.

### 4.6. Limitations and Future Directions

Although valuable results have been achieved as described above, there are still some limitations to this study. First of all, due to the age applicability of MHT, it is only suitable for left-behind children over 10 years old. This study cannot obtain the mental health data of younger children to analyze the characteristics of longitudinal changes. We recommend future researchers in this area conduct CTMA of the mental health of younger children based on other measurement tools.

In addition, most of the papers included in the analysis did not have separation duration (thus, how long a child has been separated from the parents at the time of study), but only a two-point variable: left-behind or non-left-behind. In fact, separation duration for half of the year, for 1 year and for 3 years may have different effects on mental health. In this paper, there is no way to explore the impact of separation duration on left-behind children’s mental health. In the future, we recommend that researchers further explore separation duration effect on changes in left-behind children’s mental health level and provide a more effective explanatory model for mental health changes.

## 5. Conclusions

In general, the mental health of LBC and NLBC has not changed much over the years. Compared with NLBC, LBC have poorer mental health. The mental health level of left-behind boys and girls is declining with the years, especially for left-behind boys, but the mental health level is still high. The mental health level of left-behind primary school students decreases significantly with time. However, the left-behind junior high school students do not change much. There is little difference between grades. The mental health level of LBC who lack parental supervision significantly decreases over time, while that of LBC with single-parent supervision will record little change. However, the mental health level of LBC with different guardianship types is lower than that of NLBC with both parental supervision, and there is no significant difference between the two types of LBC. 

## Figures and Tables

**Table 1:** Cross-Temporal Variable Coding Table.

Variable	Coding	Data Group 1	Sample 1	Data Group 2	Sample 2
(Non-) left-behind	1 = LBC	102	50,387	81	34,836
	2 = NLBC	81	31,782	81	31,782
Gender	1 = left-behind boys	50	11,792	50	11,792
	2 = left-behind girls	50	11,178	50	11,178
School section	1 = Elementary school	38	13,857	11	2930
	2 = Junior high school	36	10,674	11	3017
Guardianship type	1 = Single parent	22	7648	21	7103
	2 = No parental guardianship	28	11,055	21	8119

**Table 2:** Correlation results of MHT factors and age LBC and NLBC.

Index	Left-Behind Children					Non-Left-Behind Children				
	r1	r2	r3	r4	R^2^	r1	r2	r3	r4	R^2^
learning	0.048	0.045 **	0.035	0.068 **	0.002	−0.112	−0.240 **	0.161	−0.037 **	0.058
anxiety	−0.019	0.012 **	0.092	0.086 **	0.000	−0.131	−0.077 **	0.145	−0.020 *	0.006
loneliness	0.156	0.193 **	0.109	0.077 **	0.037	0.107	0.208 **	0.192	−0.002	0.043
self-blame	−0.069	−0.095 **	0.083	0.096 **	0.009	−0.158	−0.177 **	0.129	−0.051 **	0.031
allergy	−0.019	−0.010 **	0.125	0.129 **	0.000	−0.125	−0.181 **	0.224 *	0.053 **	0.033
physical	−0.108	−0.108 **	0.153	0.115 **	0.012	−0.045	−0.091 **	0.083	−0.124 **	0.008
terror	−0.068	−0.046 **	−0.034	−0.010 **	0.002	−0.126	0.000	0.099	−0.102 **	0.000
impulse	−0.050	−0.034 **	0.097	0.087 **	0.001	−0.074	0.005	0.113	−0.049 **	0.000

Note: r1 = the correlation coefficient between the unweighted age of the sample size and each factor; r2 = the correlation coefficient between the age and each factor after weighting the sample size; r3 = the initial correlation coefficient between the age and the standard deviation (SD); r4 = the correlation coefficient between the age and the standard deviation weighted according to the sample size. R^2^ = the coefficient of determination. * *p* < 0.5, ** *p* < 0.01.

**Table 3:** Changes in MHT indicators of left-behind and non-left-behind children over time.

Index	Left-Behind Children			Non-Left-Behind Children		
	ΔM	SD	$\bar{d}$	ΔM	SD	$\bar{d}$
learning	0.23	2.20	0.11	−1.51	2.18	−0.69
anxiety	0.05	1.68	0.03	−0.29	1.82	−0.16
loneliness	0.86	1.67	0.51	0.86	1.75	0.49
self-blame	−0.31	1.86	−0.17	−0.56	1.89	−0.30
allergy	−0.03	1.68	−0.02	−0.52	1.80	−0.29
physical	−0.31	2.07	−0.15	−0.29	2.17	−0.13
terror	−0.22	1.99	−0.11	0.04	2.12	0.00
impulse	−0.17	1.81	−0.09	0.03	1.91	0.01

Note: ΔM = the mean of changes in mental health over the years; SD = the standard deviation of the change of mental health level over the years; $\bar{d}$ is the effect size of changes in mental health.

**Table 4:** Differences in changes in mental health between left-behind children and non-left-behind children.

Index	Learning	Anxiety	Loneliness	Self-Blame	Allergy	Physical	Terror	Impulse
$\bar{d}$	0.27	0.26	0.29	0.17	0.22	0.24	0.24	0.24

Note: $\bar{d}$ = the effect size of changes in mental health.

**Table 5:** Changes in mental health of left-behind boys and left-behind girls over time.

Index	Left-Behind Boys			Left-Behind Girls		
	ΔM	SD	$\bar{d}$	ΔM	SD	$\bar{d}$
learning	0.83	2.31	0.36	0.79	2.30	0.34
anxiety	−0.22	1.98	−0.11	−0.18	2.59	−0.07
loneliness	0.96	1.83	0.53	1.25	1.91	0.65
self-blame	−0.03	2.02	−0.01	0.12	2.02	0.06
allergy	0.22	1.87	0.12	0.34	1.88	0.18
physical	0.40	2.27	0.18	0.04	2.28	0.02
terror	0.59	2.20	0.27	−0.33	2.30	−0.14
impulse	0.04	2.05	0.02	−0.27	2.03	−0.13

Note: ΔM = the mean of changes in mental health over the years; SD = the standard deviation of the of mental health level over the years; $\bar{d}$ = the effect size of changes in mental health.

**Table 6:** The difference in changes in mental health between left-behind boys and left-behind girls.

Index	Learning	Anxiety	Loneliness	Self-Blame	Allergy	Physical	Terror	Impulse
$\bar{d}$	−0.29	−0.18	−0.06	−0.25	0.12	−0.17	−0.45	−0.01

Note: $\bar{d}$ = the effect size of changes in mental health.

**Table 7:** Changes in mental health of left-behind elementary and junior high school students over the years.

Index	Left-Behind Elementary School Students			Left-Behind Junior High School Students		
	ΔM	SD	$\bar{d}$	ΔM	SD	$\bar{d}$
learning	−0.99	2.02	−0.49	0.05	2.37	0.02
anxiety	0.47	1.67	0.28	0.01	1.85	0.01
loneliness	1.82	1.68	1.09	0.65	1.83	0.35
self-blame	0.25	1.79	0.14	−0.99	2.25	−0.44
allergy	0.52	1.70	0.31	−0.26	1.82	−0.14
physical	0.16	2.00	0.08	−0.90	2.37	−0.38
terror	0.00	1.95	0.00	0.46	2.24	0.20
impulse	1.14	1.83	0.62	−0.17	2.02	−0.08

Note: ΔM = the mean of changes in mental health over the years; SD = the standard deviation of the change of mental health level over the years; $\bar{d}$ = the effect size of changes in mental health.

**Table 8:** Differences in mental health between left-behind primary school and junior high school students.

Index	Learning	Anxiety	Loneliness	Self-Blame	Allergy	Physical	Terror	Impulse
$\bar{d}$	−0.06	−0.08	−0.12	0.07	−0.12	−0.12	0.01	−0.28

Note: $\bar{d}$ = the effect size of changes in mental health.

**Table 9:** Changes in the mental health of left-behind children of different guardianship types over the years.

Index	Single-Parent Guardianship			Lack of Parental Guardianship		
	ΔM	SD	$\bar{d}$	ΔM	SD	$\bar{d}$
learning	0.79	1.73	0.46	1.76	1.66	1.06
anxiety	0.05	1.54	0.03	0.46	1.35	0.34
loneliness	0.21	1.57	0.13	0.98	1.36	0.72
self-blame	0.12	1.69	0.07	0.59	1.38	0.42
allergy	0.52	1.56	0.33	0.85	1.42	0.60
physical	−0.20	1.76	−0.11	0.25	1.62	0.15
terror	−0.70	1.82	−0.39	−0.68	1.43	−0.47
impulse	−0.38	1.96	−0.19	−0.05	1.61	−0.03

Note: ΔM = the mean of changes in mental health over the years; SD = the standard deviation of the change of mental health level over the years; $\bar{d}$ = the effect size of changes in mental health.

**Table 10:** Differences in mental health of left-behind children across guardianship types.

Index	Learning	Anxiety	Loneliness	Self-Blame	Allergy	Physical	Terror	Impulse
$\bar{d}$ _1_	0.107	0.19	0.18	0.02	0.13	0.12	0.15	0.13
$\bar{d}$ _2_	0.44	0.44	0.40	0.17	0.30	0.26	0.27	0.41
$\bar{d}$ _3_	0.25	0.29	0.29	0.08	0.26	0.30	0.13	0.31

$\bar{d}$_1_: the effect size of changes in mental health between LBC who lacked parental guardianship and LBC with single-parent guardianship. $\bar{d}$_2_: the effect size of changes in mental health between LBC who lacked parental guardianship and NLBC. $\bar{d}$_3_: the effect size of changes in mental health between LBC with single-parent guardianship and NLBC.

## Data Availability

Data will be made available upon request.

## References

[1] Liu X., Fan X.H., Shen J.L. (2007). Relationship between Social Support and Problem Behaviors of the Left-home-kids in Junior Middle School. Psychol. Dev. Educ..

[2] Wu Z., Qin Y. (2020). The Report of Rural Education Development in China: 2017–2018.

[3] Zhu L.J., Yan T.H., Zhang S.C., Zhang Y.L., Song Y., Li X.Y. (2020). The Relationship between Psychological Neglect and Fear of Missing Out among Left -Behind Children: The Mode rating Effect of Friendship Quality. Chin. J. Spec. Educ..

[4] Yue P.F., Hu H.Y., Fang Y. (2019). The Influence of Left-Behind Experience and Mental Resilience on Emotion Recognition. Chin. J. Spec. Educ..

[5] Tong H. (2010). A research of twenty years vicissitude: Scl-90 and its norm. Psychol. Sci..

[6] Liu X., Zhang Y., Song A., Liang Y., Qu J., Li Y., Shi J. (2013). Meta-analysis of the mental health status of left-behind children. Chin. J. Child Health Care.

[7] Li F.L., Qiao L., He J., Cheng J.Y., Xie Q.M., Zhao Q.L., Li A.-S., Li J., Guo J.-K., Yang J. (2017). Meta analysis on the Mental Health Diagnostic Test survey results of left-behind childrens in recent ten years. Chin. J. Child Health Care.

[8] Xin Z.Q., Zhang M. (2009). Changes in Chinese Middle School Students’ Mental Health (1992~2005): A Cross-Temporal Meta-Analysis. Acta Psychol. Sin..

[9] Xin S.F., Yue Y.M., Xin Z.Q., Lin C.D. (2018). Changes in Chinese Old People’s Social Support During 1996~2015: A Cross-Temporal Meta-Analysis. Psychol. Dev. Educ..

[10] Zhou B. (1993). Mental Health Diagnostic Test.

[11] Liao Y.G., Lian R. (2020). Differences in mental health changes between only children and non-only children—A cross-sectional historical study. J. Southwest Univ. (Soc. Sci. Ed.).

[12] Xin S., Liang X., Sheng L., Zhao Z. (2021). Changes of teachers’ subjective well-being in mainland China (2002~2019): The perspective of cross-temporal meta-analysis. Acta Psychol. Sin..

[13] Wang Q., Yu G.L. (2017). A Cross-Temporal Study of Lower Secondary School Students’ Mental Health. Chin. J. Spec. Educ..

[14] Chinese Ministry of Education (2013). Guidelines for Mental Health Education in Primary and Secondary Schools Revised in 2012. Ment. Health Educ. Prim. Second. Sch..

[15] General Office of the State Council of China (2018). Letter of the General Office of the State Council of China on agreeing to establish an inter-ministerial joint conference system for the care and protection of left-behind children in rural areas and the protection of children in difficulties. China Civ. Aff..

[16] State Council of China (2016). Opinions of the State Council on Strengthening the Care and Protection of Left-behind Children in Rural Areas. Gaz. State Counc. People’s Repub. China.

[17] Liu W., Yu Z.Y., Lin D.H. (2019). Resilience and Mental Health in Children and Youth: A Meta-Analysis. Stud. Psychol. Behav..

[18] Hu K. (2011). Research on the Status of Social Supports of the Rural Left-behind Children. China J. Health Psychol..

[19] Zhou Z.K., Zhao D.M., Chen J., Jiang J.C. (2003). Loneliness as a Function of Sociometric Status and Self-Perceived Social Competence in Middle Childhood. Psychol. Dev. Educ..

[20] Li Y.W. (2013). The Relation Research on Peer Attachment and Loneliness Basic on the Internet/Mobile-Phone Addiction of Adolescents. Master′s Thesis.

[21] Tian L.M., Zhang W.X., Chen G.H. (2014). Effects of Parental Support, Friendship Quality on Loneliness and Depression: To Test an Indirect Effect Model. Acta Psychol. Sin..

[22] Wang J., Zhang J., Zhu Y. (2014). Meta-analysis of the mental health of left-behind children in rural areas. Educ. Meas. Eval..

[23] Huang Y.P., Li L. (2007). Mental health status of different types of left-behind children. Chin. Ment. Health J..

[24] Wang H., Diao H., Yang L.J., Li T., Jin F., Pu Y. (2020). Relationship between adolescent knowledge-attitude-practice and resilience in left-behind children. Chin. J. Sch. Health.

[25] Liu P., Yang T.H., Wei J., Zheng Q.N. (2015). Relevant Study on Left-Behind Children’s Psychological Health and Social Support. J. Guiyang Med. Coll..

[26] Ann M., Andrew B. (2018). Resilience in Children: Developmental Perspectives. Children.

[27] Chen Y., Zhao Z. (2012). Impact of the parental rural-urban migration on the health of left-behind children in rural China. Chin. J. Healthy Policy.

[28] Liang Y.N. (2015). The Research of the Construction of Caring Association for Left-Girls in Rural Areas. Master’s Thesis.

[29] Zhang J. (2019). The Present Situation and Influencing Factors of Frustration Resistance of Upperclassmen in Primary School. Master’s Thesis.

[30] Yu H., Xia B. (2016). The mental health status of rural left-behind children in poverty-stricken areas of western China. China J. Health Psychol..

[31] Hu Y., Zhu C. (2015). A Comparative Study on the Mental Health of Rural Left-behind Children at Different School-age Stages. Soc. Sci. Hunan.

[32] Song S., Chen C., Zhang A. (2018). Effects of Parental Migration on Life Satisfaction and Academic Achievement of Left-Behind Children in Rural China—A Case Study in Hubei Province. Children.

[33] Department of Social Affairs, Ministry of Civil Affairs of China (2016). Interpretation of “Opinions of the State Council on Strengthening the Care and Protection of Left-behind Children in Rural Areas”. China Emerg. Manag..

